# “Vox Populi” Fractional Flow Reserve (vpFFR)—Leveraging Wisdom of the Crowd for the Assessment of Hemodynamic Severity of Intermediate Coronary Lesions

**DOI:** 10.3390/diagnostics16020269

**Published:** 2026-01-14

**Authors:** Natalija Odanovic, Vojko Misevic, Aleksa Obradovic, Vanja Bojic, Kosta Krupnikovic, Aleksandar Mandic, Matija Furtula, Dusan Borzanovic, Nikola Lazarevic, Stefan Zivkovic, Ivan Ilic, Milan Dobric, Samit M. Shah

**Affiliations:** 1 Institute for Cardiovascular Diseases “Dedinje”, Heroja Milana Tepica 1, 11040 Belgrade, Serbia; natalija.odanovic@yale.edu (N.O.);; 2 Section of Cardiovascular Medicine, Department of Internal Medicine, Yale School of Medicine, 333 Ceder Street, New Haven, CT 06510, USA; 3 Faculty of Medicine, University of Belgrade, Dr Subotica Starijeg 8, 11000 Belgrade, Serbia; 4 Veterans Affairs Connecticut Healthcare System, 950 Campbell Avenue, West Haven, CT 06516, USA

**Keywords:** fractional flow reserve, quantitative coronary angiography, chronic coronary syndromes

## Abstract

**Background/Objectives:** Diagnostic performance of angiography-derived physiological measures has been benchmarked against two-dimensional (2D) and three-dimensional (3D) quantitative coronary angiography (QCA), which are known for their poor correlation with hemodynamic lesion severity. Relying on the statistical concept of the wisdom of the crowd, we devised a human-performance reference for FFR surrogates, called *vox populi* FFR (vpFFR), and examined the comparative diagnostic performance of vpFFR, as well as 2D- and 3D-QCA, using invasively measured FFR as the gold standard. **Methods:** Analyses were performed in a single-center, prospective registry of consecutive FFR procedures. We calculated vpFFR as a mean of five independent, blinded predictions of the invasively measured FFR. Pearson’s correlation coefficient and receiver operating characteristic (ROC) curve analyses were used for diagnostic performance comparisons. **Results:** In 116 patients (156 vessels), Pearson’s correlation coefficients for vpFFR, 2D-, and 3D-QCA with invasively measured FFR are 0.56, −0.26, and −0.01, respectively (*p* < 0.001, *p* = 0.001 and *p* = 0.918). vpFFR has a sensitivity of 56%, specificity of 84%, positive predictive value of 67%, and negative predictive value of 76%. It correctly classified hemodynamic severity of lesions in 73% of vessels compared to 65% and 51% for 2D- and 3D-QCA, respectively. vpFFR has a larger area under the ROC curve than 2D- and 3D-QCA for predicting positive FFR (0.78, 0.63, and 0.45, respectively, *p* < 0.001). **Conclusions:** vpFFR, a mean value of five predictions of invasively measured FFR, has moderate diagnostic performance, superior to 2D- and 3D-QCA using FFR as the gold standard, and can be used as a human-performance reference for existing and emerging angiography-derived physiological measures.

## 1. Introduction

Fractional flow reserve (FFR) is the gold standard for the invasive assessment of functional significance of intermediate coronary artery stenoses, with a strong endorsement from professional societies for the management of chronic coronary syndromes [1,2,3]. However, FFR has drawbacks that limit its use in clinical practice, including the need for dedicated coronary pressure wires, administration of hyperemic agents, and, in many centers, a second invasive procedure separate from the initial diagnostic angiography [4]. To overcome these challenges, various surrogates have been developed including angiography-derived physiology software (angioFFRs) such as quantitative flow ratio (QFR, Medis), Murray-law-based quantitative flow ratio (µFR, Pulse Medical), vessel fractional flow reserve (vFFR, Pie Medical Imaging), and coronary angiography-derived fractional flow reserve (caFFR, RainMed) [5]. In broad terms, angioFFRs use computational fluid dynamics models and three-dimensional vessel reconstruction to derive physiology from angiography alone. In validation studies and when used by core laboratory users, these have been shown to have acceptable diagnostic performance, with correlation coefficients > 0.70, diagnostic accuracy of >85%, and an area under the receiver operating curve of >0.90 [6,7,8,9,10]. When assessed by an independent core lab, angioFFRs showed moderate diagnostic performance (correlation ~0.50, accuracy ~70%, AUC ~0.75), with real-world clinical utility still untested but presumed to be superior to angiographic interpretation alone [11]. Angiographic interpretation of anatomic lesion severity, as assessed visually or by two-dimensional (2D) and three-dimensional (3D) quantitative coronary angiography (QCA) as anatomic reference standards, has poor correlation with hemodynamic lesion significance, especially for intermediate severity lesions with 50–70% diameter stenosis [11,12,13].

In this study, we aimed to apply the wisdom of the crowd to estimate the hemodynamic significance of intermediate severity lesions from angiography alone. This is a statistical principle whereby a consensus from a group of individuals with diverse knowledge on the topic can accurately identify a point estimate of a continuous variable. It was first described by Aristotle and introduced in 1907 as a formal statistical concept, when the statistician Francis Galton noted that a median value of the crowd guessing the weight of an ox displayed at a stock fair lay within 1% of the actual weight of the ox [14]. This concept has been demonstrated to be accurate in many arenas, with several statistical modifications that use the mean instead of the median as the *vox populi* guess [15,16,17,18,19].

Using the principle of wisdom of the crowd, we derived a novel index for hemodynamic significance assessment, called *vox populi* FFR (vpFFR), and compared its diagnostic performance with respect to invasively measured FFR against two-dimensional and three-dimensional quantitative coronary angiography (2D- and 3D-QCA).

## 2. Materials and Methods

### 2.1. Patient Selection

Patients were included from “Dedinje FFR/QFR Registry”, a single-center prospective registry of consecutive patients who underwent a clinically indicated cardiac catheterization and wire-based FFR measurement at the Institute for Cardiovascular Diseases “Dedinje” (NCT06659367). Patients had either previously undergone a diagnostic angiography and had a Heart Team decision to undergo FFR measurement of one or more vessels, or FFR measurement was performed ad hoc immediately after diagnostic angiography. Patients were eligible for registry inclusion if they were older than 18 years and if they provided informed consent to be included in the registry. Patients were ineligible if they did not undergo a successful FFR measurement (e.g., planned FFR but the procedure was canceled, technical issues precluded FFR measurement, etc.) and if they were unable or unwilling to provide informed consent to be included in the registry. The written informed consent for registry inclusion was obtained upon procedure completion to avoid a perception of duress. This study was approved by the Ethical Board of the Institute for Cardiovascular Diseases “Dedinje” (approval number 4669, date of approval 16 September 2024), and it was conducted in accordance with the Declaration of Helsinki.

### 2.2. FFR Measurements

The participants underwent wire-based FFR measurement of at least one major epicardial coronary artery with adenosine hyperemia. Access site was radial or femoral and either a 6F or a 7F sheath was used. Patients were given 70–100 U/kg of heparin for anticoagulation. A 6F guiding catheter was used to engage the coronary artery in question. The shape of the guiding catheter and the size of the guiding catheter curve were left to the operator’s discretion. Extensive flushing of the system prior to pressure calibration was strongly encouraged. Several different pressure wires were used in this study: PressureWire X (Abbott Vascular, Redwood Park, CA, USA), OmniWire (Philips, Amsterdam, The Netherlands), Verrata (Philips, Amsterdam, The Netherlands), Comet II (Boston Scientific, Marlborough, MA, USA), and OptoWire (OpSens, Quebec City, QC, Canada). Intracoronary nitroglycerin administration prior to FFR measurement, as well as guiding catheter flushing with saline prior to pressure equalization in the aorta, was strongly encouraged. Adenosine was administered either intracoronary (recommended dose 100 mcg for the right coronary artery or 200 mcg for the left coronary artery system) or intravenously (at the dose of 140 mcg/kg/min). The choice of pressure wire manufacturer and route of adenosine administration (intracoronary versus intravenous) were left to the operator’s discretion. FFR value was reported as the lowest ratio of Pd to Pa during stable hyperemia. Where several measurements of FFR were performed, the lowest value was designated as the value of FFR. Each procedure was concluded by the drift check. A drift of >0.03 was considered significant and required remeasurement of FFR.

### 2.3. vpFFR Derivation

Five interventional cardiologists or fellows in training, blinded to the patients’ clinical data and actual FFR results, were asked to predict vessel FFR based on angiography alone. The guessers were shown the patient’s angiography (with age and gender) and told that the patient was going to have (or already had) FFR measurement of a particular blood vessel or vessels. All available diagnostic images were provided to the guessers as well as FFR wire position where available. The guessers independently provided their predictions by noting them down on a piece of paper and handing them over before they could discuss the angiogram with any other guessers or make remarks. We then calculated vpFFR as the arithmetic mean of their predictions. Interventional cardiologists were considered to be early career if they had been practicing interventional cardiology for less or equal to 5 years, and experienced if they had been practicing for more than 5 years. The guessers did not receive any training or calibration, and each only had their clinical experience with performing FFR to rely on when making the guess.

### 2.4. QCA

A certified operator performed 2D- and 3D-QCA by using Medis Suite (Medis Medical Imaging Systems, Leiden, The Netherlands). The operator was blinded to the FFR or vpFFR results. Briefly, for 2D-QCA all diagnostic images were loaded into the software, and an optimal view that displays clearly the vessel and lesion(s) of interest was selected for the analysis. For 3D-QCA, two optimal non-foreshortened views of the vessel at least 25 degrees apart were used. The automatic pathline and contour detection were corrected where appropriate. The automatic reference diameter was used as the default setting, unless the operator felt the need to define the reference diameter by the normal vessel areas or to use a fixed proximal diameter (e.g., in cases of ostial or proximal disease). For 3D-QCA, morphology tab displaying the minimum and maximum vessel diameter along the pathline was used for quality control. If there was a large discrepancy between the minimum and maximum diameters, the operator would recheck the contours at those sites.

### 2.5. Statistical Analysis

Nominal and ordinal variables are shown as counts (% in parenthesis) and continuous variables as means (standard deviation in parenthesis). Pearson’s correlation coefficient is used to describe the correlation of vpFFR, 2D-QCA, and 3D-QCA with FFR, and receiver operating characteristic (ROC) curve analysis is used to compare the diagnostic performances of the three methods. To compare the differences in correlation coefficients between the two groups, we transformed absolute correlation coefficients to Z scores and used Fisher’s exact test. To determine interclass correlation coefficient between FFR and vpFFR, as well as between different raters, we used a two-way random model and absolute agreement type. Bland–Altman analysis was used to assess overall bias, limits of agreement, and proportional bias. Assuming a type 1 error of 5%, in a population where prevalence of positive FFR is ~33%, 129 samples would be needed to detect 50% sensitivity, and 64 samples would be needed to detect 50% specificity (with a 15% marginal error). We therefore decided that at least 130 vessels needed to be included in the analysis. All analyses were performed using SPSS version 26, and *p*-value < 0.05 was considered statistically significant.

## 3. Results

**Figure 1:**
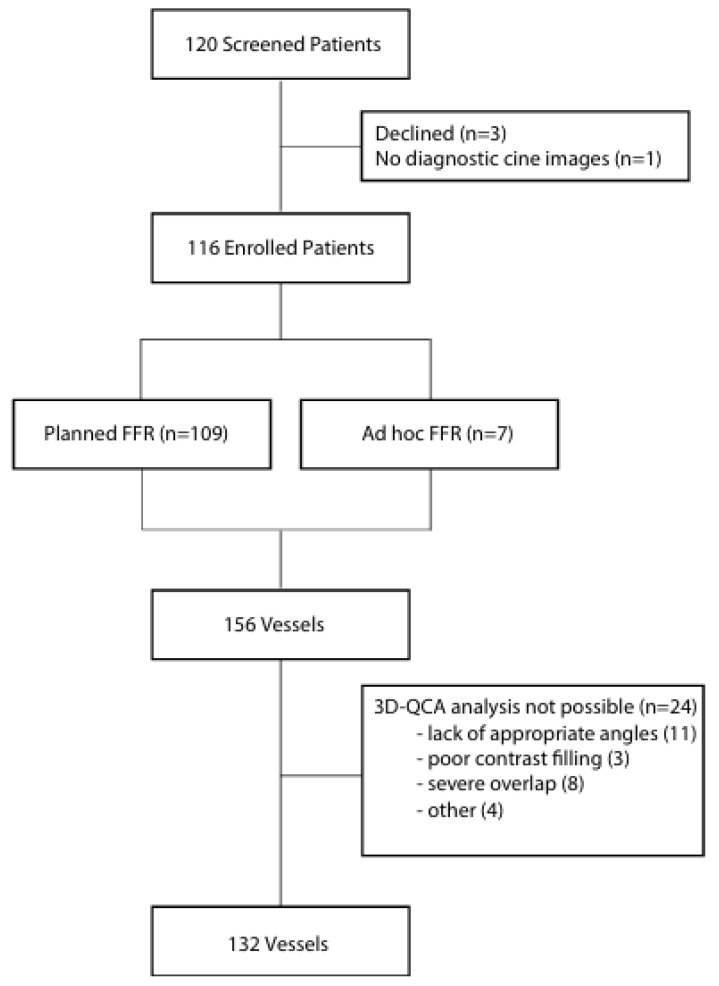
Patient inclusion diagram.

Of the first 120 patients screened between October 2024 and December 2024, three declined to participate and one was missing data, so 116 patients (156 vessels) were included in the analysis (Figure 1). Most FFR procedures (94%) were planned, and few were ad hoc (6%).

vpFFR and 2D-QCA were obtained in 100% (*n* = 156) of vessels, while 3D-QCA was obtained in 84.6% (*n* = 132) of vessels. The most common reasons for the inability to obtain 3D-QCA were lack of appropriate angles (46% of cases), followed by severe vessel overlap (33% of cases).

Demographic characteristics of participants, risk factors, comorbidities, symptoms, and vessel characteristics are summarized in Table 1. Patients were mostly male (72.4%), with a high prevalence of risk factors. Prior revascularization with either percutaneous coronary intervention (PCI) or coronary artery bypass grafting surgery (CABG) was present in 35.4% of patients. The most commonly interrogated vessel was the left anterior descending (LAD) artery (59.6%), followed by the right coronary artery (RCA) (22.4%) and left circumflex artery (LCx) (16.7%). Most patients had no (37.1%) or mild anginal symptoms (Canadian Cardiovascular Society angina grade I or II in 38.8% of patients). Average FFR was 0.83 ± 0.08, and average diameter stenosis by 2D-QCA was 46% ± 9%. Using a cutoff of ≤0.80, 37.8% of patients had a positive FFR.

vpFFR operator characteristics and individual statistics are shown in Table 2. Overall, 370 (47.4%) guesses came from fellows in training, 200 (25.6%) from early career interventional cardiologists, and 210 (26.9%) from experienced interventional cardiologists.

**Figure 2:**
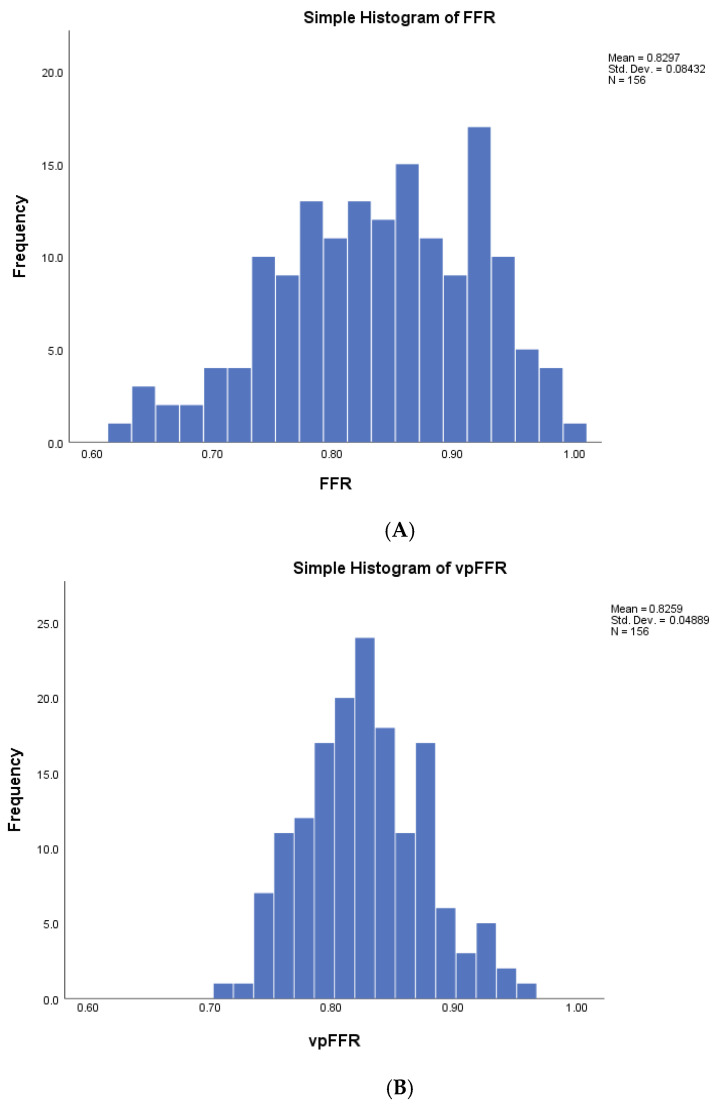
Distributions of FFR and vpFFR. Histogram displaying the distribution of invasively measured FFR (**A**) and vpFFR (**B**). FFR = fractional flow reserve; vpFFR = vox populi fractional flow reserve.

Average vpFFR was 0.83 ± 0.05, and it was less widely distributed than FFR (Figure 2).

### 3.1. Diagnostic Performance of vpFFR, 2D-, and 3D-QCA

**Figure 3:**
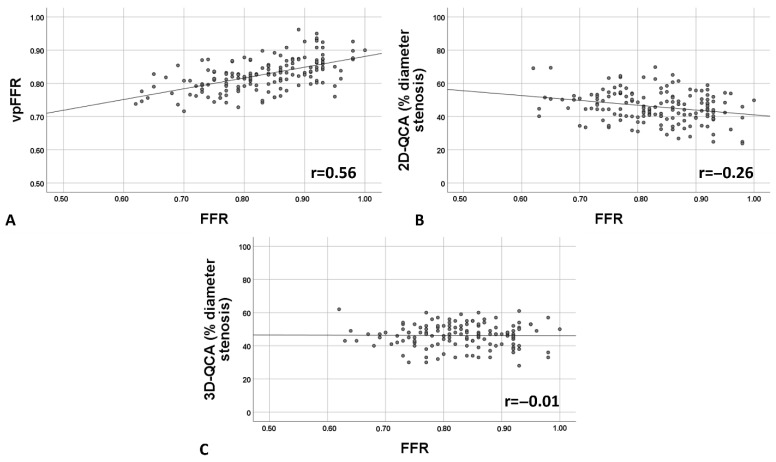
Scatter plot for correlation of vpFFR, 2D-QCA, and 3D-QCA with FFR. Scatter plot displaying correlation of vpFFR (**A**) of 2D-QCA (**B**) and 3D-QCA (**C**) with invasively measured FFR. 2D-QCA = 2-dimensional quantitative coronary angiography; 3D-QCA = 3-dimensional quantitative coronary angiography; FFR = fractional flow reserve; vpFFR = vox populi fractional flow reserve.

**Figure 4:**
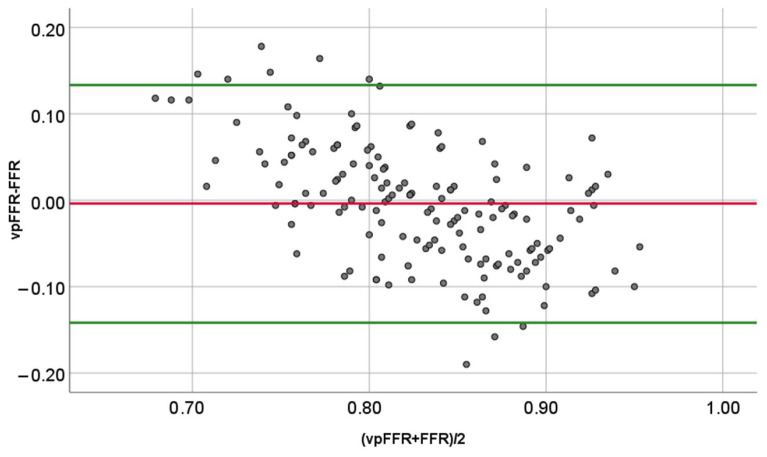
Bland–Altman analysis of vpFFR and FFR agreement. FFR = fractional flow reserve; vpFFR = vox populi fractional flow reserve.

vpFFR showed moderate correlation with wire-based FFR (r = 0.56, *p* < 0.001), which was superior to 2D- (r = −0.26, *p* = 0.001) and 3D-QCA (r = −0.01, *p* = 0.918) correlation coefficients (Figure 3, *p* = 0.001 and *p* < 0.001 for the respective differences). The interclass correlation coefficient between FFR and vpFFR was 0.654 (95% confidence interval 0.526–0.748, *p* < 0.01), indicating moderate agreement. A Bland–Altman analysis (Figure 4) revealed that, while there was no systematic bias (mean difference −0.00379), there was a substantial proportional bias: at lower values of FFR, vpFFR tended to overestimate it, and at higher values of FFR, it tended to underestimate it.

For a binary FFR threshold of >0.80 or ≤0.80, diagnostic accuracy of vpFFR was 73% compared to 65% and 51% for 2D- and 3D-QCA, respectively (Table 3).

**Figure 5:**
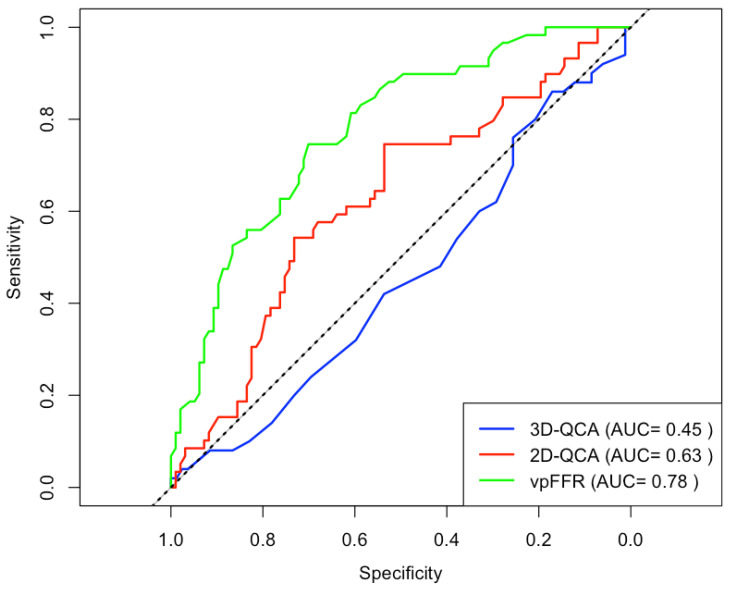
ROC curve analysis for vpFFR, 2D-, and 3D-QCA. ROC curves for vpFFR, 2D-QCA, and 3D-QCA using invasively measured FFR (cutoffs ≤0.80 and >0.80) as a reference. 2D-QCA = 2-dimensional quantitative coronary angiography; 3D-QCA = 3-dimensional quantitative coronary angiography; AUC = area under the curve; FFR = fractional flow reserve; ROC = receiver operating characteristic; vpFFR = vox populi fractional flow reserve.

vpFFR has a significantly larger area under the ROC curve (0.78) than both 2D- (0.63) and 3D-QCA (0.45) (*p* < 0.001 and *p* = 0.003, respectively) (Figure 5). Based on ROC curve analysis, the optimal cutoff value for vpFFR is 0.82, which results in a sensitivity of 75%, specificity of 70%, PPV 60%, and NPV 82%.

### 3.2. The Influence of Operator Experience on vpFFR Accuracy

Early career interventional cardiologists had the highest correlation coefficient at 0.61 (*p* < 0.001), significantly higher than fellows in training (0.34, *p* < 0.001) (*p* < 0.001 for the difference, Table 4), as well as the highest area under the ROC curve (0.84) compared to both experienced interventional cardiologists (0.72, *p* = 0.09 for the difference) and fellows in training (0.67, *p* < 0.001 for the difference). All five operators were in binary agreement in 44% of the cases, and four out of five operators agreed in 28% of the cases. Diagnostic accuracy of binary vpFFR was 84% when all operators were in agreement and 53% when only three operators agreed (Table 5). Overall, the operators had an interclass correlation coefficient of 0.870 (95% confidence interval 0.835–0.900, *p* < 0.01), indicating strong agreement.

### 3.3. The Influence of Vessel Type on vpFFR Accuracy

The summary of diagnostic performance of vpFFR depending on vessel type is shown in Table 6. Binary diagnostic accuracy of vpFFR is the best for the left circumflex coronary artery (LCX) and the worst for the left anterior descending artery (LAD).

## 4. Discussion

We present a human-performance reference for FFR surrogates, called *vox populi* FFR or vpFFR, and demonstrat its superiority over anatomic assessment with 2D- and 3D-QCA for predicting hemodynamic lesion significance using wire-based FFR as the gold standard. These results quantify the instinctive interpretation of intermediate severity lesions and demonstrate the performance of vpFFR, which appears to be comparable to commercially available angiography-derived physiology software.

The concept of the wisdom of the crowd in the context of hemodynamic lesion assessment was previously used in a study by Foley et al., who asked 25 interventional cardiologists with an average of 6.8 ± 4.9 years of experience to predict FFR in 200 patients from the Objective Randomized Blinded Investigation with optimal medical Therapy of Angioplasty in Stable Angina (ORBITA) trial [20]. Vessels included in the ORBITA study had angiographic stenosis severity > 70% (average diameter stenosis by QCA 64%), which is not representative of the intermediate 50–70% diameter stenoses that are normally referred for physiologic assessment, as are the ones included in our study (average diameter stenosis by QCA 46%). This translated into physiologically higher lesion severity than in our study (median FFR 0.72 versus 0.84 in our study). Foley et al. found that visual assessments of FFR were more narrowly distributed than the invasively measured FFR, which is also demonstrated in our study. Finally, they found a correlation coefficient of 0.69 between invasively measured FFR and “vFFR-group”—a variable representing the mean value of FFR guesses for a particular vessel, which is the conceptual equivalent of vpFFR. The higher correlation coefficient in their study is explained by two main factors: the higher number of guesses used to obtain the mean value and operator experience. In our study, almost 50% of the guesses came from fellows in training, whose guesses had a lower diagnostic performance than those from early career and experienced interventional cardiologists. We also observed an inverted U-shaped relationship between vpFFR operator experience and guess accuracy (lowest for fellows, best for early career, intermediate for experienced), which may be explained by the greater adoption of wire-based physiology among younger generations of practicing interventional cardiologists. Furthermore, a more careful selection of vpFFR guessers or a larger sample could potentially yield even higher diagnostic performance for vpFFR. In our study, in order to keep the vpFFR guess feasible, we arbitrarily chose five participants to review angiograms and allowed for a large number of guesses to be made by fellows in training. Had we chosen 10, 20, or more participants, or selected the high-performing FFR guessers to make all predictions, the diagnostic performance of vpFFR would likely have been even higher as in the study by Foley et al., but the study may have been less feasible.

Importantly, the correlation of vpFFR with invasive FFR and its diagnostic performance were comparable to the correlation and diagnostic performance of five different angioFFR software when tested by an independent academic core laboratory [11]. The derivation of vpFFR is not intended to inform clinical practice, but rather to serve as a comparator for these emerging FFR surrogates. We provide a methodical demonstration that an educated guess of a group of physicians can outperform anatomic measurements and raise the diagnostic performance bar for angiography-derived FFR surrogates.

This dataset reflects clinical practice and is well aligned with prior datasets used to examine the diagnostic performances of angioFFRs with some distinctions. The ratio of male patients, the distribution of blood vessels, and patient symptoms are consistent with the datasets that examined the diagnostic accuracy of angioFFRs. Similarly, the average FFR and the percentage of positive FFRs reflect the existing literature; however, the standard deviation of FFR is slightly lower (0.08) than in the studies of angioFFRs (~0.12), indicating lower variance and truly intermediate lesions closer to the cutoff point. This may have led to an even lower diagnostic performance of vpFFR than the one that would be expected in a dataset with wider variance [21,22]. The presence of a proportional bias on Bland–Altman analysis is consistent with the narrower distribution of vpFFR versus FFR, as noted on the histograms (Figure 2), and reflects the genuine clinical uncertainty regarding the hemodynamic significance of intermediate lesions.

### Limitations

The limitations of the study are that it was based on a single-center registry, where all anatomic and hemodynamic analyses were performed by clinical users rather than an independent core laboratory. All assessments were performed per vessel and not per lesion. Institute for Cardiovascular Diseases “Dedinje” is a high-volume center for FFR procedures, providing vpFFR guessers with ample clinical experience and limiting the generalizability of vpFFR predictions. The choice of five guessers is arbitrary, and the method was not validated internally or externally. While it is known that intra-observer variability of some angiography-derived physiology software is high, this was not tested for vpFFR due to feasibility reasons [23]. Since the number and experience of guessers can lead to variable diagnostic performance of vpFFR, further multicenter studies, with different numbers of guessers and guessers of different levels of experience are needed to test the robustness, reproducibility, and generalizability of the method.

## 5. Conclusions

In conclusion, vpFFR represents the quantification of an educated guess for the prediction of hemodynamic significance of intermediate severity coronary stenoses and serves as a human-performance reference for angiography-derived surrogates of wire-based FFR.

## Figures and Tables

**Table 1:** Demographic characteristics, risk factors, comorbidities, symptoms, and vessel characteristics.

	Total (*n* = 116)
Age	65.3 (±8.5)
Sex	
Female	32 (27.6%)
Male	84 (72.4%)
BMI (average)	27.5 (±4.3)
Hypertension	115 (99.1%)
Hyperlipidemia	111 (95.7%)
Diabetes mellitus	49 (42.2%)
Smoking status	
Never smoker	47 (40.5%)
Former smoker	34 (29.3%)
Current smoker	35 (30.2%)
Family history of CAD	54 (46.6%)
Prior revascularization of CAD	
Prior PCI	40 (34.5%)
Prior CABG	1 (0.9%)
PAD	16 (13.8%)
CVA	13 (11.1%)
CKD	12 (10.3%)
HFrEF (EF < 40%)	20 (17.2%)
Symptoms	
None	43 (37.1%)
CCS I	22 (19.0%)
CCS II	23 (19.8%)
CCS III	13 (11.2%)
CCS IV	4 (3.4%)
Dyspnea	11 (9.5%)
Vessels	
LAD or branches	93 (59.6%)
LCX or branches	26 (16.7%)
RCA or branches	35 (22.4%)
RI or branches	2 (1.3%)
FFR	0.83 ± 0.08
≤0.80	59 (37.8%)
>0.80	97 (62.2%)
Diameter stenosis by 2D-QCA	46% ± 9%
≥0.50	54 (34.6%)
<0.50	102 (65.4%)

2D-QCA = 2-dimensional quantitative coronary angiography; BMI = body mass index; CABG = coronary artery bypass grafting; CAD = coronary artery disease; CCS = Canadian Cardiovascular Society; CKD = chronic kidney disease; CVA = cerebrovascular accident; FFR = fractional flow reserve; HFrEF = heart failure with reduced ejection fraction; LAD = left anterior descending artery; LCX = left circumflex artery; PAD = peripheral artery disease; PCI = percutaneous coronary intervention; RCA = right coronary artery; RI = ramus intermedius.

**Table 2:** vpFFR individual operator characteristics and statistics.

Operator ID	Operator Experience/ Level of Training	Number of Guesses	Mean FFR in the Subgroup	Standard Deviation of Mean FFR	Mean Guess	Standard Deviation of Mean Guess	Pearson’s Correlation Coefficient	*p* Value (One Tailed)
1	Fellow	114	0.83	0.09	0.82	0.05	0.36	<0.001
2	Fellow	21	0.81	0.09	0.82	0.06	0.23	0.153
3	Fellow	26	0.82	0.08	0.80	0.04	0.07	0.377
4	Fellow	77	0.83	0.08	0.83	0.06	0.35	0.001
5	Fellow	22	0.83	0.09	0.83	0.05	0.41	0.029
6	Fellow	12	0.82	0.10	0.82	0.05	0.26	0.210
7	Fellow	6	0.81	0.11	0.81	0.06	0.93	0.004
8	Early Career IC	21	0.83	0.08	0.80	0.07	0.61	0.002
9	Fellow	29	0.82	0.07	0.82	0.05	0.18	0.180
10	Fellow	63	0.83	0.09	0.81	0.06	0.43	<0.001
11	Early Career IC	124	0.83	0.08	0.84	0.06	0.60	<0.001
12	Experienced IC	6	0.83	0.09	0.82	0.10	0.90	0.007
13	Experienced IC	76	0.84	0.08	0.83	0.06	0.31	0.003
14	Experienced IC	14	0.82	0.09	0.82	0.05	0.66	0.005
15	Experienced IC	4	0.87	0.11	0.83	0.10	0.88	0.058
16	Experienced IC	15	0.82	0.08	0.81	0.04	0.33	0.116
17	Experienced IC	41	0.82	0.09	0.83	0.07	0.57	<0.001
18	Experienced IC	53	0.82	0.09	0.82	0.05	0.53	<0.001
19	Experienced IC	1	0.71	-	0.80	-	-	-
20	Early Career IC	55	0.84	0.08	0.84	0.07	0.68	<0.001

Fellow = fellow in training; Early Career IC = early career interventional cardiologist (within 5 years of fellowship completion); Experienced IC = experienced interventional cardiologist (beyond 5 years of fellowship completion).

**Table 3:** Diagnostic performance of vpFFR, 2D-QCA, and 3D-QCA.

	vpFFR (*n* = 156)	2D-QCA (*n* = 156)	3D-QCA (*n* = 132)
Sensitivity	55.9%	49.2%	28.0%
Specificity	83.5%	74.2%	64.6%
PPV	67.3%	53.7%	32.5%
NPV	75.7%	70.6%	59.5%
Diagnostic accuracy	73.0%	64.7%	50.7%
AUC	0.777	0.625	0.454
Pearson correlation coefficient	0.560	−0.262	−0.010
*p* value for Pearson correlation coefficient	<0.001	0.001	0.915

AUC = area under the curve; NPV = negative predictive value; PPV = positive predictive value.

**Table 4:** Diagnostic performance of vpFFR depending on operator experience.

Vessel	Number of Vessels	Pearson’s Correlation Coefficient	*p* Value for Pearson’s Correlation Coefficient (One-Tailed)	Binary Diagnostic Accuracy	Area Under ROC Curve
Fellow	370	0.34	<0.001	63.5%	0.67
Early career IC	200	0.61 *	<0.001	75.0%	0.84 * ^¥^
Experienced IC	210	0.48	<0.001	65.7%	0.72

* *p* < 0.05 relative to the “Fellow” group; ^¥^ *p* < 0.05 relative to the “Experienced IC” group; Fellow = fellow in training; Early Career IC = early career interventional cardiologist (≤5 years of practice); Experienced IC = experienced interventional cardiologist (>5 years of practice).

**Table 5:** Diagnostic performance of 5-, 4-, and 3-operator binary agreement.

	5-Operator Agreement (*n* = 68)	4-Operator Agreement (*n* = 43)	3-Operator Agreement (*n* = 45)
Sensitivity	61.9%	52.6%	31.6%
Specificity	93.6%	70.8%	69.2%
PPV	81.3%	58.8%	42.9%
NPV	84.6%	65.4%	58.1%
Diagnostic accuracy	83.8%	62.8%	53.3%

NPV = negative predictive value; PPV = positive predictive value.

**Table 6:** Correlation of vpFFR depending on vessel type.

Vessel	Number of Vessels	Pearson’s Correlation Coefficient	*p* Value for Pearson’s Correlation Coefficient (One-Tailed)	Binary Diagnostic Accuracy	Area Under ROC Curve
LAD	93	0.46	<0.001	66.7%	0.71
LCx	26	0.22	0.140	84.6%	0.69
RCA	35	0.48	0.002	80.0%	0.75
RI	2	-	-	100%	-

LAD = left anterior descending artery; LCx = left circumflex artery; RCA = right coronary artery; RI = ramus intermedius; vpFFR = vox populi fractional flow reserve.

## Data Availability

The raw data supporting the conclusions of this article will be made available by the authors on request.

## Abbreviations

The following abbreviations are used in this manuscript:

FFR	Fractional Flow Reserve
ORBITA	The Objective Randomized Blinded Investigation with optimal medical Therapy of Angioplasty in Stable Angina
ROC	Receiver Operating Characteristic
QCA	Quantitative Coronary Angiography
vpFFR	vox populi Fractional Flow Reserve
